# Deciphering the divergent transcriptomic landscapes of cervical cancer cells grown in 3D and 2D cell culture systems

**DOI:** 10.3389/fcell.2024.1413882

**Published:** 2024-08-13

**Authors:** Roshan Kumar, Marissa Iden, Shirng-Wern Tsaih, Rachel Schmidt, Akinyemi I. Ojesina, Janet S. Rader

**Affiliations:** ^1^ Department of Obstetrics and Gynecology, Medical College of Wisconsin, Milwaukee, WI, United States; ^2^ Medical College of Wisconsin Cancer Center, Milwaukee, WI, United States; ^3^ Post-Graduate Department of Zoology, Magadh University, Bodh Gaya, Bihar, India

**Keywords:** cervical cancer, SiHa cell line, 3 dimensional, 2 dimensional, HPV

## Abstract

Cervical cancer remains a significant health challenge for women worldwide, with a disproportionate impact on developing regions like sub-Saharan Africa. Taking advantage of recent advancements in developing suitable preclinical models to study cell proliferation, differentiation, and gene expression, we used RNA sequencing to compare the transcriptomic profiles of SiHa cervical cancer cells grown in 3D versus 2D culture systems. Pathway analysis of 3D cultures revealed upregulation of immune activation, angiogenesis, and tissue remodeling pathways. The high expression of cytokines, chemokines, matrix metalloproteinases, and immediate early genes, suggests that 3D cultures replicate the tumor microenvironment better than 2D monolayer cultures. HPV gene expression analysis further demonstrated higher expression levels of HPV16 *E1*, *E2*, *E6*, and *E7* genes in 3D versus 2D cultures. Further, by using a set of linear models, we identified 79 significantly differentially expressed genes in 3D culture compared to 2D culture conditions, independent of HPV16 viral gene effects. We subsequently validated five of these genes at the protein level in both the SiHa cell line and a newly developed, patient-derived cervical cancer cell line. In addition, correlation analysis identified 26 human genes positively correlated with viral genes across 2D and 3D culture conditions. The top five 3D versus 2D differentially expressed and HPV-correlated genes were validated via qRT-PCR in our patient derived cell line. Altogether, these findings suggest that 3D cultures provide superior model systems to explore mechanisms of immune evasion, cancer progression and antiviral therapeutics.

## Introduction

Simple, large scale *in vitro* cell culture systems are critical for developing and testing new therapeutics for invasive cervical cancer (ICC). A major barrier in researching ICC therapeutic targets has been the lack of appropriate, yet simple, preclinical models that better recapitulate *in vivo* cell to cell contacts of the solid tumor. Our understanding of the development, progression, and treatment of ICC has been significantly advanced via use of model systems, especially immortalized cell lines (zur Hausen, 2002; Pinto et al., 2020; Iden et al., 2021). While reproducible, traditional two-dimensional (2D) cell cultures fail to mimic the complex tumor architecture and interactions found *in vivo* (Kapalczynska et al., 2018; Fontoura et al., 2020). These interactions are vital for mimicking *in vivo* tumor growth; influencing processes like cell proliferation, differentiation, and expression of genes and proteins important for cancer progression (Fontoura et al., 2020; Shi et al., 2022).

In recent years, three-dimensional (3D) spheroid and organoid cultures have emerged as promising *in vitro* model systems for screening anti-cancer drugs against different cancer types (Melissaridou et al., 2019; Fontoura et al., 2020; Jensen and Teng, 2020; Pinto et al., 2020; Shi et al., 2022; Bromma et al., 2023). Spheroid cultures exhibit several features that more closely mimic avascular tumor nodules, including 3D architecture, extensive cell-cell interactions, diffusion barriers leading to spatial heterogeneity in nutrient and oxygen levels, as well as regions of quiescence and hypoxia, and metabolic heterogeneity - all of which better recapitulate the tumor microenvironment (TME) of solid tumors compared to conventional 2D monolayer cultures (Duval et al., 2017; Kapalczynska et al., 2018; Fontoura et al., 2020). These attributes confer enhanced resistance to many anti-cancer drugs compared to 2D monolayer cultures, thus allowing for studies of processes such as proliferation, invasion, angiogenesis, and drug response in a context better reflecting the human tumor (Kapalczynska et al., 2018; Melissaridou et al., 2019; Tanaka et al., 2021).

Here, we characterized transcriptomes from HPV16^+^ SiHa cervical cancer cells grown in a 3D, non-scaffold, spheroid culture system and conventional 2D monolayer culture. Differential gene expression analysis revealed upregulation of genes associated with immune activation and inflammation in the 3D system. Likewise, genes linked to tissue development and cell differentiation were downregulated in the 3D system. Interestingly, we observed higher HPV16 gene expression in 3D versus 2D cultures and subsequently identified a set of host genes positively correlated with HPV viral gene expression. We also identified a distinct group of differentially expressed genes in 3D cultures which were independent of viral gene expression. The expression pattern of genes highlighted in the 3D model recapitulated an inflammatory TME phenotype and viral oncogene expression closely mimicking the complex biology of the *in vivo* tumor niche. Finally, these findings were validated at the mRNA and protein level in a novel, patient-derived cervical cancer cell line.

## Materials and methods

### Cell culture

The SiHa cervical cancer cell line was obtained from the American Type Culture Collection (ATCC; HTB35™) and maintained in Dulbecco’s Minimum Essential Medium (DMEM, ThermoFisher) with 10% Fetal Bovine Serum (FBS, Omega Scientific). MCW-3 is a HPV45^+^, patient-derived cervical cancer cell line generated in our laboratory using standard techniques. Briefly, the patient tumor was washed in 1X PBS and minced into smaller pieces that were further dissociated by incubating in KSFM (Invitrogen, ThermoFisher) containing 2.0 units/mL dispase (Fisher Scientific), 3X gentamicin (ThermoFisher), 3X penicillin/streptomycin (ThermoFisher), and 1X fungizone (ThermoFisher) overnight at 4°C. The following day, tumor pieces were placed in KSFM containing 0.25% Trypsin-EDTA (ThermoFisher) and digested on ice for 30 min with occasional agitation. Next, tumor pieces in KSFM/Trypsin-EDTA were incubated at 37°C for 10 min, followed by addition of 3 mL FBS and centrifugation at 500 RPM for 5 min. After centrifugation, the supernatant was carefully removed, and cells were resuspended and plated at high density in F-medium (3:1 [v/v] F-12 [Ham]-DMEM (Invitrogen, ThermoFisher), 5% FBS, 0.4 μg/mL hydrocortisone (Millipore Sigma), 5 μg/mL insulin (Millipore Sigma), 8.4 ng/mL cholera toxin (Millipore Sigma), 10 ng/mL EGF (R&D Systems), 24 μg/mL adenine (Millipore Sigma), 100 U/mL penicillin, and 100 μg/mL streptomycin) containing 10 µM ROCK inhibitor, Y-27632 (Selleck Chemicals). Cells were slowly transitioned into F-media without ROCK inhibitor after 10 passages. All cultures were kept at 37°C in a high-humidity environment with a gas mixture consisting of 95% air and 5% CO_2_. Cell line authenticity was verified through STR profiling (ATCC), and cells were regularly checked for *mycoplasma* using the MycoStrip™ detection kit (InvivoGen). Cell harvesting was performed using a 0.25% Trypsin-EDTA solution and cell counting carried out using the Countess-3 Automated Cell Counter (Invitrogen) according to the manufacturer’s protocol.

All SiHa cells were cultured in DMEM supplemented with 10% FBS, whereas MCW-3 cells were cultured in F-media without ROCK inhibitor, as described above. For 2D culture experiments, 5 mL (2 x 10^5^ cells/mL) of SiHa or MCW-3 cells were seeded into 10 cm dishes, then overlayed with an additional 5 mL of their respective growth media. For 3D cultures, 5,000 cells were seeded in each well of a 96-well Nunclon Sphera, U-bottom plate (ThermoFisher), then centrifuged at 70 × g for 5 min before placing in the incubator. Media replacement was performed every 2–3 days, and spheroid formation was monitored for 7–9 days.

### RNA extraction, sequencing, and analysis

The 2D monolayer cultures for SiHa included three distinct cell passages, with 2 replicates per passage. The 2D biological replicates were generated in 2 separate batches: passage 1 replicates (2D_P1_R1, 2D_P1_R2) in Batch 1 and passage 2 and 3 replicates (2D_P2_R1, 2D_P2_R2, 2D_P3_R1, 2D_P3_R2) in Batch 2 (Supplementary Figure S1). For 3D spheroid SiHa cultures, 3 biological replicate samples were generated (3D_R1, 3D_R2, 3D_R3) separately. Total RNA was extracted using the PureLink™ RNA Mini Kit (ThermoFisher) as per manufacturer’s protocol, including on-column DNase treatment. The purity and concentration of isolated total RNA was assessed using the NanoDrop^®^ ND-1000 spectrophotometer (ThermoFisher). RNA was shipped overnight on dry ice to Novogene Co., LTD. (Sacramento, CA) for library preparation and 150 bp, paired-end RNA sequencing on the Illumina NovaSeq 6000.

Raw sequencing reads were evaluated for quality control using FastQC v0.11.9 (https://www.bioinformatics.babraham.ac.uk/projects/fastqc/). Metrics assessed included adapter content, read length distribution, and per-base sequence quality scores. A hybrid human-virus reference genome was created by concatenating the human primary assembly (GRCh38) with HPV16 (NC_001526.4) and HPV45 (X74479.1) genome assemblies. Reads were aligned to the custom, hybrid human-virus reference genome using the STAR v2.7.10b aligner (Dobin et al., 2013). Transcript/gene-level expression abundance was quantified from aligned reads using RSEM v1.3.3 (Li and Dewey, 2011). Genes with raw counts >10 in at least 3 samples were considered expressed and retained for further analysis. To comprehensively assess sample similarity within the experimental design, Poisson Distance was computed using the CRAN package PoiClaClu version 1.0.2.1 (https://CRAN.R-project.org/package=PoiClaClu). Principal Component Analysis (PCA) plots were generated using rlog-transformed values to visualize group relationships.

In this study we analyzed two subsets of differentially expressed genes (DEGs). First, we examined the overall DEGs between the 3D and 2D systems using a baseline model (M1). Going beyond this overall analysis, we aimed to identify genes whose expression differences between 3D and 2D conditions were consistent and unaffected by the expression of HPV16 viral genes. For all the subsets, DE analysis was performed using the Bioconductor package DESeq2 version 1.36.0 (Love et al., 2014) to compute log_2_FoldChange (L2FC) and False Discovery Rate (FDR) adjusted *P*-values (*P-adj*). Statistical significance was determined at an FDR threshold of 0.05.

### Functional analysis

To analyze functional attributes of DEGs, we selected genes with absolute |L2FC| ≥ 1.5 and *p-adj* < 0.05 as statistically significant. Unless stated otherwise, all genes included in the subsequent analyses were selected based on these criteria. Pathway analysis and construction of a protein-protein interaction (PPI) network were then performed on selected DEGs using Ingenuity Pathway Analysis software (IPA; QIAGEN). Pathways or networks with an absolute *Z*-score ≥ 2 and *p-adj* < 0.05 were considered significant.

GO enrichment analysis for baseline model (M1) was conducted using the Bioconductor package DOSE version 3.22.0 (Yu et al., 2015). We applied the Benjamini-Hochberg (BH) method for multiple-testing correction. Pathways with adjusted *p*-value < 0.05 were considered significant. To assess the similarity between enriched terms, pairwise term similarity was calculated, and results visualized as an enrichment map (emapplot; https://bioconductor.riken.jp/packages/3.6/bioc/html/clusterProfiler.html).

### Identifying genes specific to 3D versus 2D culture conditions

To identify these genes, a series of linear models were fitted to analyze differential gene expression patterns between 3D and 2D culture conditions. Initially, model 1 (M1), which considered only culture condition, was used and DE analysis was performed using DESeq2 version 1.36.0 (Love et al., 2014), yielding baseline expression differences attributable to the 3D vs. 2D conditions. Subsequently, four additional models (Models 2–5) were fitted, each incorporating the “culture condition” factor alongside the normalized expression of a specific HPV16 viral gene (*E6*, *E7*, *E2*, or *E1*) as covariates. This approach was used to identify potential modulatory effects of individual HPV16 viral genes on the DE patterns observed in M1. Genes consistently identified as DE across M1, and all subsequent models (Models 2–5), represent a set likely influenced primarily by the 3D-2D culture condition independent of HPV16 viral gene expression. A heatmap was plotted for all those genes exhibiting absolute |L2FC| ≥ 1.5 and *p-adj* < 0.05. The functional analysis for this set of genes was performed using IPA, following the methodology described in the preceding section.

### Analysis of HPV gene expression patterns

HPV genes with RSEM expected counts ≤10 in fewer than 3 samples were filtered out, leaving only *E1, E2, E6* and *E7*. Hierarchical clustering of a combined matrix of human and HPV genes was performed to identify groups of genes with similar expression patterns across samples. RSEM count was log transformed and clustered using Pearson correlation and average linkage method. Initial clustering was performed by generating multiple numbers of clusters while aiming to achieve a higher level of clustering resolution among HPV genes. The process involved incrementing the number of clusters to the point at which one or more of the HPV genes began to segregate from a singular cluster. Upon setting the number of clusters to *k* = 157, the genes *E1*, *E2*, *E6*, and *E7* remained grouped within a single cluster (designated as cluster 6; Supplementary Table S1A). *E7* formed an individual cluster, distinct from *E1*, *E2*, and *E6* when the cluster count increased to 158. Pearson correlation matrix was generated using the clustered genes including the HPV genes, to visualize consensus gene expression patterns.

We also curated a comprehensive list of genes encoding proteins reported to interact with *E1* (Castro-Munoz et al., 2019), *E2* (Muller and Demeret, 2012), *E6* (White et al., 2012) and/or *E7* (Fischer et al., 2017; Supplementary Table S1B). Genes from unsupervised clustering (Supplementary Table S1A) were compared to the curated list of reported HPV-interacting genes from the literature (Supplementary Table S1B). The common twenty-six genes on both lists were visualized using box plots to show distribution of expression and bar plots for comparison of L2FC between genes.

### Quantitative real time PCR

RNA was extracted from MCW-3 cells as described above for RNAseq. cDNA was synthesized using the High-Capacity RNA-to-cDNA Kit (Applied Biosystems), as per manufacturer’s instructions. Expression was assessed using iTaq universal SYBR Green Supermix (BioRad; Hercules, CA) on a CFX Connect Real-Time PCR Detection System (BioRad). The thermal cycling program included an enzyme activation step at 95°C for 10 min, followed by 40 cycles of a 10 s denaturing step at 95°C, and a 1 min annealing/extension step at 60°C. Fluorescent intensity was measured at 60°C or 62°C at the end of each cycle. We analyzed the expressions of five human genes (*ANKZF1*, *NOS3*, S100A9, SERPINB4, and *IGFBP3*) selected from the 26 consensus genes correlated with HPV gene expression in addition to HPV45 oncogenes (*E6*, *E7*, and *E6*/*E7*). Primers for *RPS18* were used for data normalization. Primer sequences are listed in Supplementary Table S2.

### 3D and 2D protein extraction and western blotting

Cells from 2D monolayer cultures (3.6 × 10^6^) or spheroids (started from 5,000 cells; 192 spheroids pooled) were washed with 1X PBS and incubated in RIPA cell extraction buffer (ThermoFisher) supplemented with 1X Halt Protease and Phosphatase inhibitor cocktail (ThermoFisher) at −20°C overnight. The following day, protein lysates were thawed on ice and homogenized by running through a 21G needle. Undigested cellular debris was pelleted by centrifugation at 10,000 RPM for 5 min at 4°C and protein was quantified using a standard Bradford Assay (Sigma). Cell lysate (30 μg) was run on a Criterion Tris-HCl polyacrylamide gel (Bio-Rad), transferred to PVDF membranes, blocked with 1X TBS + 10% nonfat dry milk at room temperature for 1 h, and incubated with primary antibody overnight at 4°C. The following day, membranes were washed, incubated for 1 h at room temperature with HRP-conjugated goat anti-rabbit or mouse anti-rabbit IgG (Cell Signaling Technology; Danvers, MA), and developed using ECL (Life Technologies). The following primary antibodies were purchased from Cell Signaling Technology: ALPP (#8681), IL-18 (#67775), S100A9 (#72590), Claudin-1 (#13995), and Caspase-14 (#36809). Chemiluminescence was visualized using an iBright Imaging System (ThermoFisher) and visualized protein bands were quantified using the iBright imaging software.

## Results

### 3D spheroid morphology, transcriptome sequencing, and data analysis

We performed RNA sequencing to evaluate the transcriptomic landscape of SiHa cervical cancer cells grown in 3D versus 2D culture conditions. The SiHa 3D spheroids exhibited an average diameter of 249.23 ± 11.4 µm (mean ± sd). A total of 6 replicate samples were originally sequenced for 2D monolayer cultures, while 3 replicate samples underwent sequencing for 3D cultures. After quality control and clustering analysis, one 2D replicate (2D_P2_R1) was excluded from comparative analysis, leaving a total of 8 samples for subsequent downstream DE analysis (n = 5 for 2D and n = 3 for 3D). On average, 3D cultures generated approximately 25,691,160 reads (±336,564), while 2D cultures produced 28,128,011 reads (±6,401,246).

**FIGURE 1:**
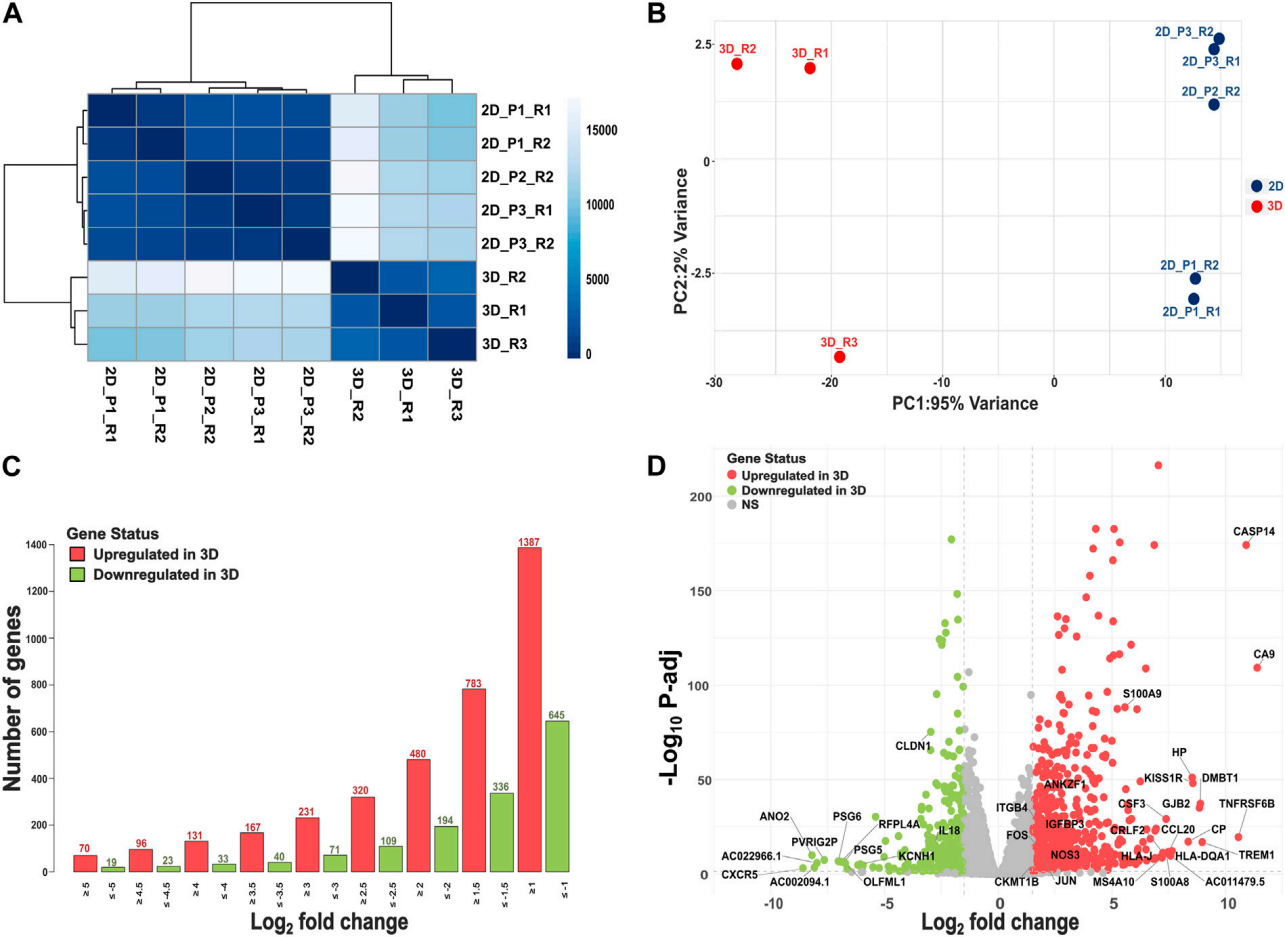
Overview of RNAseq analysis. **(A)** A heatmap depicting similarities and dissimilarities between samples, calculated using the Poisson distance method. **(B)** PCA plot illustrating group differences, with data transformed using the rlog method. **(C)** Depiction of the number of DEGs, both upregulated (L2FC > 1.5, FDR ≤ 0.05) and downregulated (L2FC < −1.5, FDR ≤ 0.05), at different log2 fold change values. **(D)** Volcano plot showing results of DE analysis in 3D versus 2D culture conditions.

Using the Poisson distance method, we observed all 3D and 2D samples formed separate clusters (Figure 1A). Principal coordinate analysis further highlighted a clear separation between 3D and 2D culture systems (Figure 1B). By integrating multiple 2D passages and sequencing them in separate batches, our experimental design was aimed to minimize batch effects and capture the underlying biological variability between 3D and 2D cultures. Out of the significant DEGs using baseline model (M1), 783 exhibited higher expression in 3D cultures (L2FC > 1.5, FDR ≤ 0.05), while 336 genes displayed lower expression in 3D versus 2D culture (L2FC < −1.5, FDR ≤ 0.05). Figure 1C illustrates the number of genes that exhibited significantly higher or lower expression in 3D cultures at various L2FC thresholds. Even with a L2FC cutoff of ±5, we observed a total of 70 significantly upregulated genes and 19 significantly downregulated genes in 3D cultures. The top 10 significantly upregulated genes and their (respective L2FC) in SiHa 3D cell cultures were: *CA9* (11.39), *CASP14* (10.90), *TNFRSF6B* (10.56), *TREM1* (8.97), *DMBT1* (8.89), *GJB2* (8.85), *KISS1R* (8.56), *HP* (8.53), *CP* (8.35), and *HLA-DQA1* (7.64; Figure 1D; Supplementary Table S3). Likewise, the top 10 significantly downregulated genes in the 3D condition were *CXCR5* (−8.59), *ANO2* (−8.19), AC002094.1 (−8.09), AC022966.1 (−7.97), *PVRIG2P* (−7.65), *PSG6* (−7.03), *RFPL4A* (−6.92), *PSG5* (−6.77), *OLFML1* (−6.73), and AC011500.3 (−6.65; Figure 1D; Supplementary Table S3).

### Functional analysis

Ingenuity Pathway Analysis on overall significant DEGs in 3D versus 2D SiHa cultures identified upregulation of pathogen-induced cytokine storm and wound healing pathways in 3D systems (Supplementary Figure S2). These pathways shared several DEGs, including cytokines (*IL1B*, *IL1A*, *IL11*, *IL33*, *CXCL8*, *TNFSF10,* and *TNFSF14*), collagenases (*COL13A1* and *COL27A1*), TNF receptor (*TNFRSF1B*) and transcription factors (*FOS* and *JUN*). The pathogen-induced cytokine storm pathway also exhibited increased expression of various cytokines (*IL17C*, *CCL20*, *CCL28*, *CSF2*), chemokines (*CXCL1*, *CXCL2*, *CXCL3*, *CXCL10*), collagenases (*COL6A1*, *COL6A2*, *COL7A1*, *COL9A2*, *COL9A3*), and HLA receptor components (*HLA-DMA*, *HLA-DMB*, *HLA-DPA1*, *HLA-DPB1*, *HLA-DQA1*, *HLA-DRA*, *HLA-DRB1*, *HLA-DRB5*). Additionally, the wound healing signaling pathway showed increased expression of numerous other cytokines and growth factors, including *IL17C*, *CSF2*, *LIF*, *PDGFB*, and *VEGFA*. Extracellular matrix (ECM) components including various collagen genes and fibronectin (*FN1*) were induced along with matrix metalloproteases (*MMP10* and *MMP1*). Collectively, pathway analysis revealed upregulation of inflammatory mediators, growth factors, ECM components, and proteases involved in promoting proliferation, migration, angiogenesis, tissue remodeling, and wound healing in 3D cultures.

Immune activation was a hallmark of 3D cells with upregulation of multiple interconnected pathways, including macrophage classical activation, IL-17A signaling, acute phase response, antigen presentation, and TME pathways. Notably, the TME pathway exhibited increased expression of key genes such as *CSF2*, *CSF3*, *CXCL8*, and *IL1B*; matrix metalloproteinases like *MMP1*, *MMP3*, *MMP10*, and *MMP17*; and transcriptional regulators *FOS* and *JUN*. Additionally, the analysis revealed downregulation of the cell cycle mediator, *CCND1*, and upregulation of the NF-κB subunit, *RELB*. Further, 3 growth factors associated with the TME pathway, *FGF11*, *PDGFB*, and *VEGFA*, displayed increased expression in 3D cells. These 3D SiHa cell expression patterns clearly suggest activation of inflammatory mediators, ECM remodeling enzymes, specific transcriptional regulators, and growth factors associated with the TME pathway.

Gene Ontology (GO) enrichment analysis further confirmed significant upregulation of biological processes related to immune activation and inflammation (Supplementary Figure S3; Supplementary Table S4A), including peptide antigen assembly and MHC class II complex formation, antimicrobial and antibacterial humoral responses, leukocyte adhesion, peptidase regulation, and generalized inflammatory signaling. Angiogenesis, response to growth factor, and apoptotic signaling pathway were the top abundant biological processes in 3D cultures. Further, the GO molecular function analysis revealed 3D culture upregulation of endopeptidase and peptidase inhibitory activities, endopeptidase/peptidase regulator activities, signaling receptor activator/ligand activities, and molecular transducer/signaling receptor activities (Supplementary Figure S3; Supplementary Table S4B). Downregulated molecular functions in 3D versus 2D cultures were related to glycosaminoglycan, growth factor, and sulfur compound binding, as well as ECM structural constituents, actin binding, and structural molecule activities.

Downregulation of multiple pregnancy-specific glycoproteins (PSGs) highlights a key difference between 3D and 2D SiHa cultures. PSGs are members of the immunoglobulin superfamily and produced by placental trophoblasts and dysregulated in cancer (Moore et al., 2022). The 3D culture model showed decreased expression of PSGs compared to the 2D model. Five PSG genes (*PSG1*, *PSG2*, *PSG5*, *PSG6*, and *PSG9*) were significantly downregulated in 3D (L2FC ranging from −3.08 to −7.03).

### DEGs specific to culture dimension and independent of HPV

**FIGURE 2:**
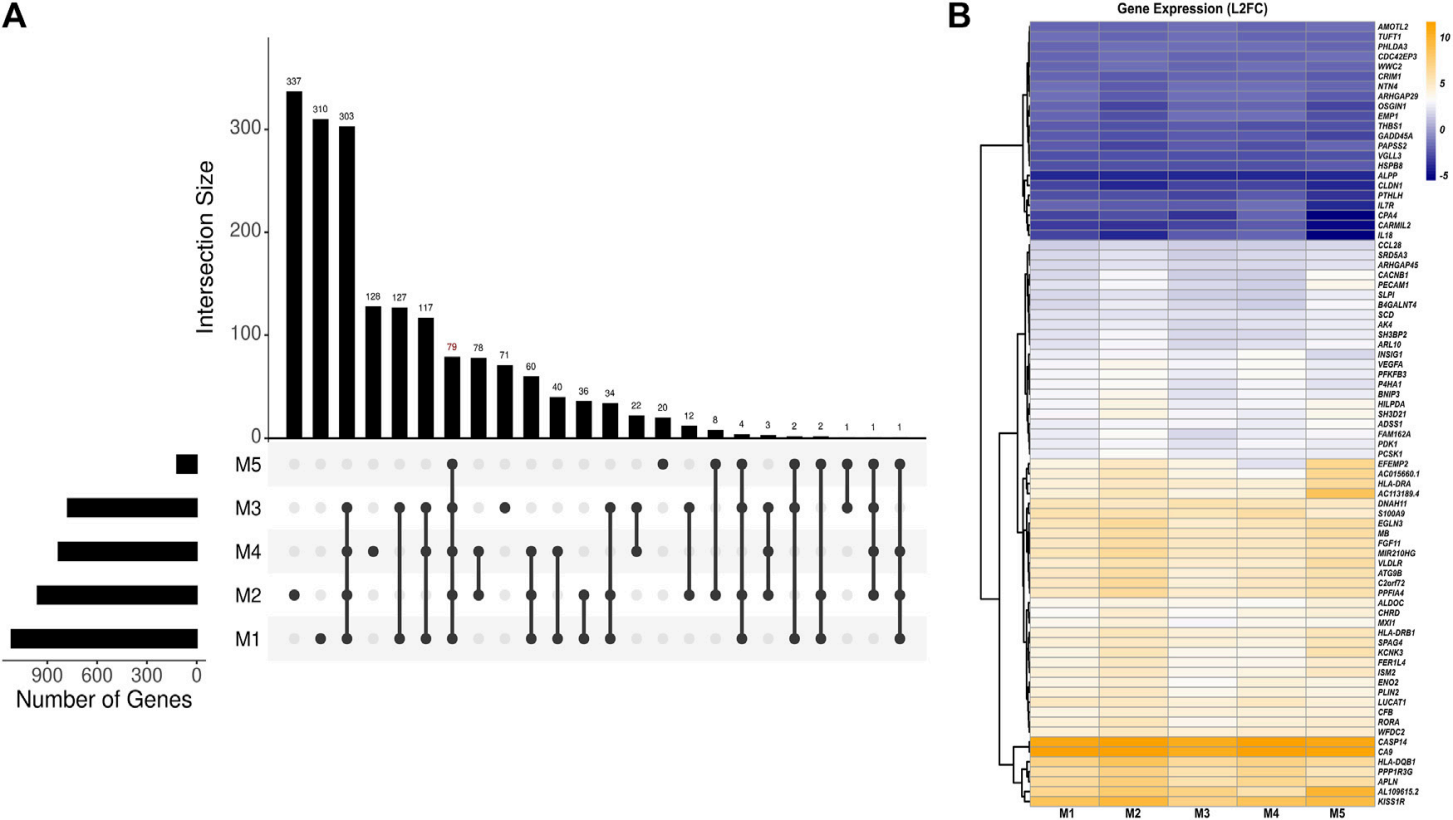
Analysis of HPV-independent DEGs in 3D versus 2D SiHa cultures. **(A)** UpSet plot depicting number of key DEGs between 3D and 2D cell culture conditions across five linear models (M1: Y ∼ culture_condition, M2: Y ∼ HPV16_E6 + culture_condition, M3: Y ∼ HPV16_E7 + culture_condition, M4: Y ∼ HPV16_E2 + culture_condition, M5: Y ∼ HPV16_E1 + culture_condition). The top panel displays the number of DEGs in various model combinations (intersections). The bottom panel summarizes the relationships among the models by highlighting shared and unique DEGs identified by each model. **(B)** Heatmap depicting 79 DEGs across models suggesting their expression changes are likely specific to the cell culture condition (M1) and independent of HPV16 viral gene expression.

Using a set of linear models, we identified a total of 79 DEGs in 3D versus 2D culture conditions independent of the effects of HPV16 viral gene expression (Figures 2A,B; Supplementary Table S5). Among these, 57 genes were upregulated, and 22 genes were downregulated in 3D versus 2D cultures. The top five upregulated genes were *CA9*, *CASP14*, *KISS1R*, *HLA-DQB1*, and AL109615, while the top five downregulated genes were A*LPP*, *CARMIL2*, *CPA4*, *CLDN1*, and *IL-18*. Of note, proinflammatory protein S100A9 was associated with 3D-2D culture conditions. Pathway analysis of these DEGs revealed upregulation of immune response genes in 3D culture. Notably, these included genes involved in crucial immune processes such as neutrophil degranulation (*ALDOC*, *ARHGAP45*, *PECAM1*, *S100A9*, and *SLPI*) and T-cell receptor signaling (*HLA-DQB1*, *HLA-DRA*, *HLA-DRB1*, and *PDK1*). Top predicted activated upstream regulators included dimethyl n-oxalyl-glycine (hypoxia-inducible factor-proline dioxygenase inhibitor), *HIF1A* (Hypoxia Inducible Factor 1 Subunit Alpha), cobalt chloride (hypoxia-mimetic agent), and *IOX2* (potent HIF-1α prolyl hydroxylase-2 (PHD2) inhibitor), while the inhibited upstream regulators included *EGLN1* (Egl-9 Family Hypoxia Inducible Factor 1). Activated HIF and inhibited *EGLN1* regulators suggests a more hypoxic environment in 3D spheroids which may better mimic the known hypoxic environment of HPV+related cervical cancers.

### HPV and correlated host gene expression in 3D vs. 2D

**FIGURE 3:**
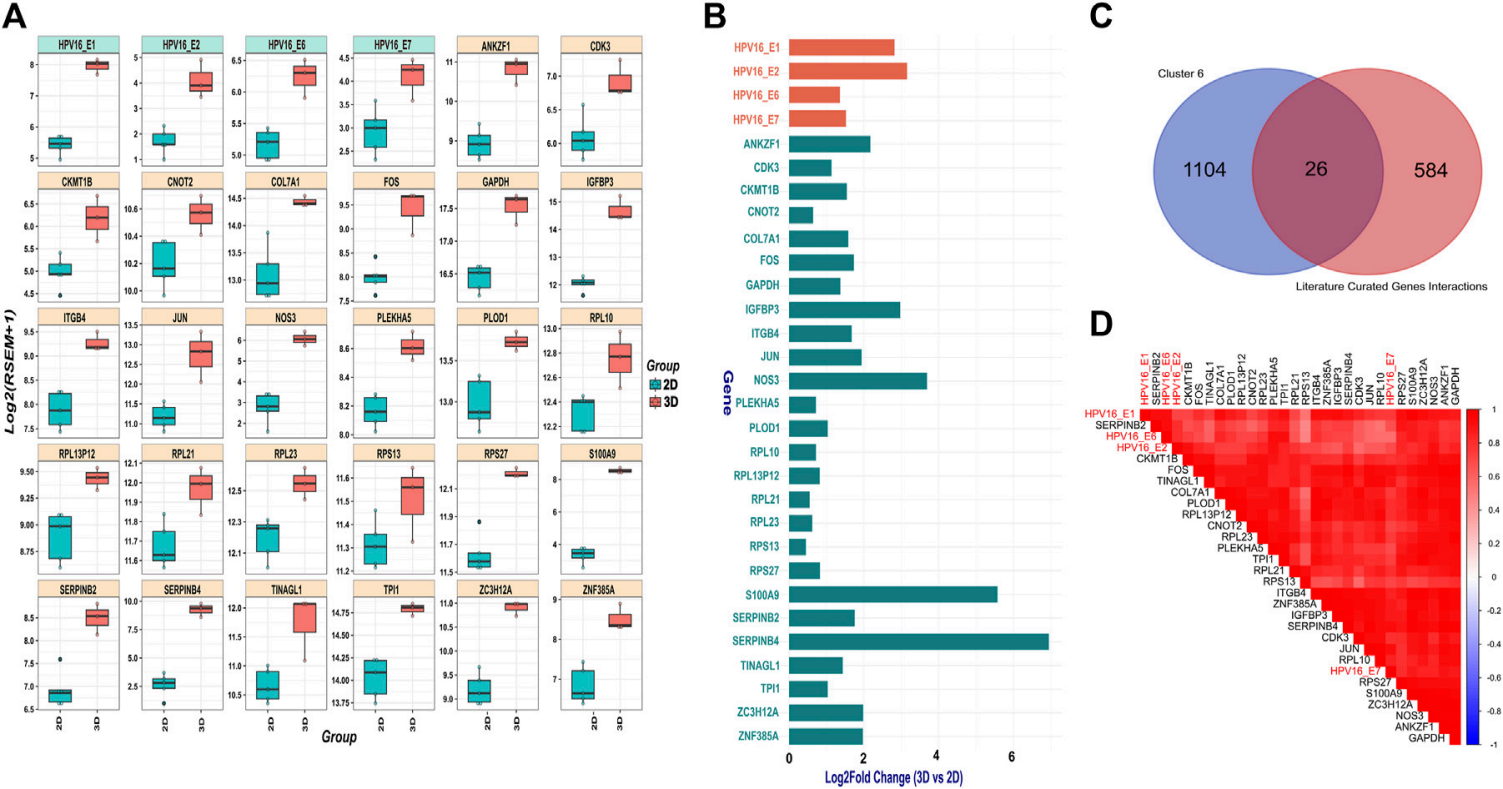
HPV gene and correlated host gene expression in 3D vs. 2D SiHa cultures **(A)** Boxplots showing distribution of 26 host genes and HPV16 gene expression across 3D and 2D SiHa cultures. **(B)** Barplot comparing L2FC in expression of the 26 DEGs correlated with HPV gene expression. **(C)** Venn diagram showing overlap of the 26 human genes identified by Pearson correlation and hierarchical clustering (*n* = 1,156) and literature-curated HPV-associated genes (*n* = 636). **(D)** Correlation plot depicting higher correlation of the 26 selected host genes with HPV16 gene expression.

SiHa cells harbor integrated HPV16 within 13q22, with the 3’integration site precisely located within intron 2 of the noncoding *LINC00393* gene. Our analysis revealed higher expression of HPV16 *E1*, *E2*, *E6* and *E7* genes in 3D versus 2D SiHa (Figures 3A,B). Then, we used the Pearson correlation method to identify 1,130 human genes from cluster 6 that were highly positively correlated with HPV16 gene expression. Next, we compared these correlated genes to a literature-curated list of 610 genes known to be associated with HPV16 *E1*, *E2*, *E6* and *E7* genes (Supplementary Table S1B). We identified 26 human genes common between the two sets. We then examined expression of these 26 genes in 3D versus 2D SiHa cell cultures. All 26 genes were significantly increased in 3D compared to conventional 2D cultures, while 13 genes exhibited a L2FC greater than 1.5 (Supplementary Table S6; Figures 3A–D). Moreover, linear model analysis revealed that the expression of these genes was significantly impacted by viral gene expression (Supplementary Table S6), suggesting that 3D cell culture systems may better recapitulate HPV-host gene regulatory dynamics. Among these genes are key players in cell cycle regulation and cancer (*CDK3* and *COL7A1*), upregulation of transmembrane receptor involved in tumorigenesis and invasiveness (*ITGB4*); (Li et al., 2017), and genes involved in inflammation and tissue remodeling (*S100A9 and PLOD1*); (Rader et al., 2008; Lim et al., 2016; Qi and Xu, 2018). Interestingly, S100A9 is correlated with HPV genes but also significantly expressed in our 3D-2D culture associated genes analysis, suggesting its expression appears to be independent of direct regulation by HPV16 viral genes. Further, large ribosomal subunit genes (*RPL10*, *RPL23*, *RPL13P12*, *RPL21*), small ribosomal subunit genes (*RPS27* and *RPS13*), and AP-1 transcription factors (*JUN* and *FOS*) exhibited increased expression in 3D cultures. Other genes that displayed positive correlation to HPV gene expression in 3D SiHa cells included *IGFBP3*, *ANKZF1*, *CNOT2*, *ZNF385A*, *NOS3*, *ZC3H12A*, *CKMT1B*, *TINAGL1*, *GAPDH*, *TPI1*, and *PLEKHA5*.

### Validation of top HPV-correlated and 3D differentially expressed genes

**FIGURE 4:**
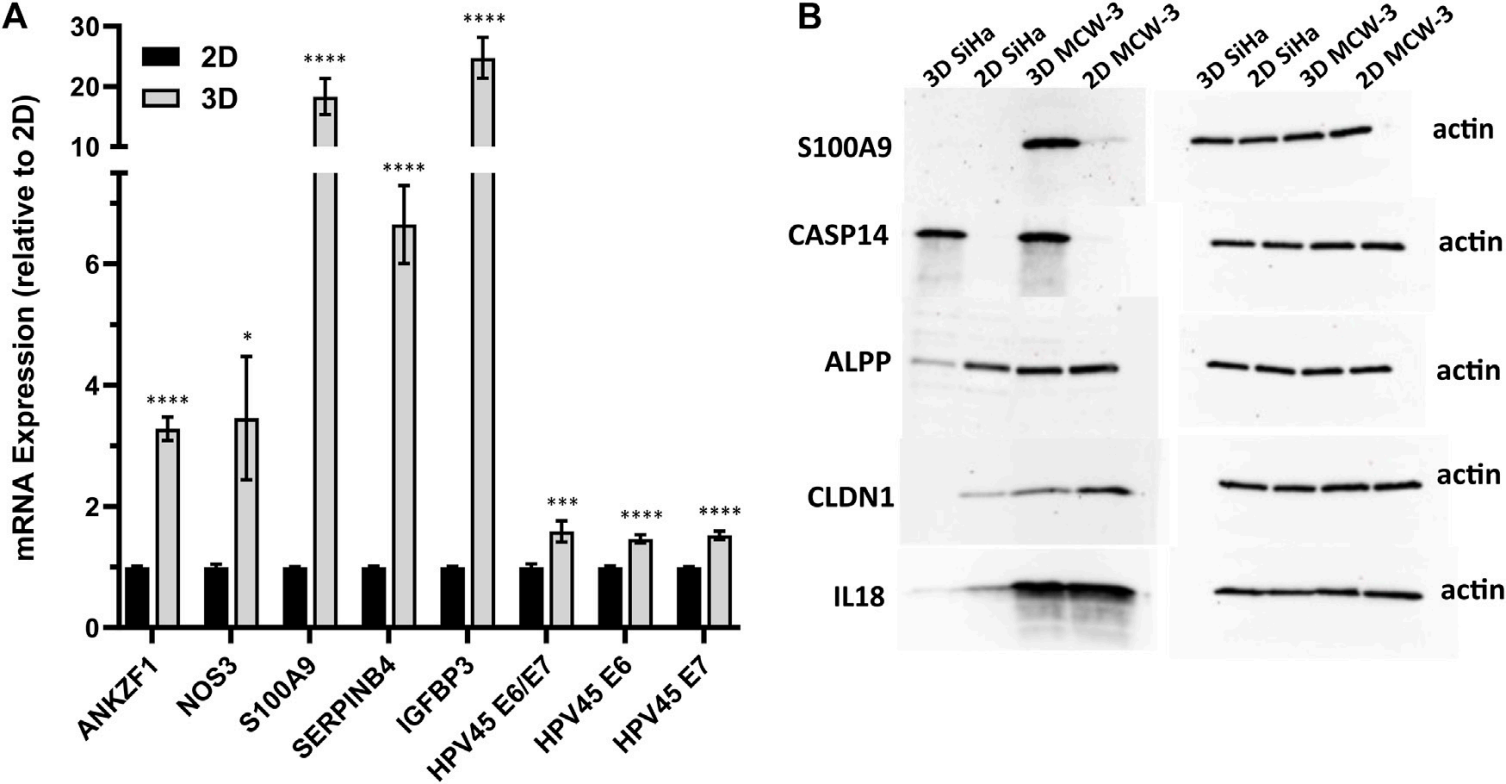
Validation of key host gene expression in 3D vs. 2D cultures. **(A)** RT-qPCR validation of top DEGs and HPV45 genes in HPV45+ primary cervical cancer cells (MCW-3) grown in 3D vs. 2D culture. Results shown as mean fold change in 3D relative to 2D (error bars: SE, *n* = 3 biological x 3 technical replicates). *p*-value < 0.01*, <0.0001***, <0.00001 ****; **(B)** Western blot analysis of HPV-independent genes, *CASP14*, *S100A9*, *ALPP*, *CLDN1*, and *IL18*, with differential expression in 2D-3D cultures.

We next sought to validate the top HPV-correlated and 3D culture-associated DE genes in an HPV45^+^, primary cervical tumor-derived cell line (MCW-3). The MCW-3 spheroids had an average diameter of 294.0 ± 7.67 µm (mean ± sd). As depicted in Figures 4, 3D MCW-3 cultures displayed significantly higher expression of *ANKZF1*, *NOS3*, *S100A9*, *SERPINB4*, *IGFBP3*, and HPV45 oncogenes, *E6* and *E7* in comparison to 2D cultures. Thus, elevated expression of these host and viral genes appears to be translatable across cervical cancer cells and different HPV types at the transcriptomics level.

To further validate the 3D-2D culture associated genes at the protein level, we performed Western blot analysis on a subset of top upregulated/downregulated genes: *CASP14* (Caspase 14, L2FC = 10.9), *S100A9* (S100 Calcium Binding Protein A9, 14, L2FC = 5.5), *ALPP* (Alkaline Phosphatase, Placental, L2FC = −4.0), *CLDN1* (Claudin-1, L2FC = −3.0), and *IL18* (Interleukin 18, L2FC = −2.8) (Figure 4B). Notably, *CASP14*, which exhibited significant upregulation in the 3D transcriptomic data, demonstrated consistent protein-level elevation in Western blot analysis of 3D cultures. Interestingly, although the *S100A9* gene was upregulated in the 3D SiHa transcriptomics data and the corresponding protein followed a similar pattern in MCW-3, *S100A9* protein was not detected in SiHa western blots (Figure 4B). Conversely, *ALPP*, *CLDN1*, and *IL18*, all of which were transcriptomically downregulated in 3D cultures, showed higher protein abundance in their respective 2D controls, closely mirroring mRNA expression patterns.

## Discussion

Transcriptomic analysis revealed significant differences in gene expression in 3D compared to 2D cervical cancer cell cultures, with genes associated with inflammation, immune activation, angiogenesis, hypoxia, and tissue remodeling being upregulated in 3D culture. These findings align with previous studies demonstrating similar gene expression patterns in 3D matrix cultures compared to 2D cultures (DelNero et al., 2015; Liu et al., 2021). Notably, our study highlights that even a simple spheroid model, without the use of scaffolds or matrices, can recapitulate these characteristic changes in gene expression associated with 3D tumor microenvironments. Consistent with the findings of DelNero et al. (2015), who employed alginate hydrogel scaffolds, our scaffold-free 3D spheroid model exhibited upregulation of genes associated with extracellular matrix remodeling (e.g., *MMP1*, *MMP10*, *LOX*), angiogenesis (e.g., *CXCL1*), and hypoxia response (e.g., *CA9*, *EGLN3*). This suggests that even a simple spheroid model can recapitulate gene expression patterns characteristic of the tumor microenvironment, without the need for exogenous scaffolds or matrices. Genes associated with epithelial cell differentiation, tissue morphogenesis, and response to growth factors were downregulated in 3D culture relative to 2D culture. In 3D cultures, we also observed higher HPV viral gene expression. Consequently, correlation analysis was conducted to identify host genes highly correlated with HPV gene expression. The top 5 HPV-correlated human genes, which were also upregulated in the 3D environment, were further validated via RT-qPCR in a newly established, patient-derived cervical cancer cell line, confirming specific upregulation within 3D cervical cancer cultures. We also identified gene sets exhibiting significant differential expression in 3D versus 2D culture conditions, irrespective of whether the HPV16 genes (*E6*, *E7*, *E2*, *E1*) were included in the differential expressed model or not. Thus, differential expression of these genes is robust and unaffected by the presence of HPV viral gene expression.

In recent years, a transition towards adoption of 3D cell culture systems has occurred as accumulating evidence suggests they provide a more physiologically relevant representation of the TME compared to 2D monolayers, thereby yielding critical biological insights (Duval et al., 2017; Fontoura et al., 2020; Jensen and Teng, 2020; Bromma et al., 2023). The primary goal of this study was to enable systematic comparisons between the transcriptome of 3D versus traditional 2D monolayer cultures. The overall pathway analysis between 3D and 2D cultures revealed enrichment of pathways associated with pathogen-induced cytokine storm, wound healing, and the tumor microenvironment (TME). This enrichment suggests that the 3D SiHa transcriptome more closely resembles the signaling underlying inflammation, extracellular matrix (ECM) remodeling, angiogenesis, and proliferation observed in *in vivo* cervical tumors (Cook et al., 2022; Yuan et al., 2023). Higher expression of cytokines like *IL1*, *CSF2*, and *IL33*, chemokines such as *CXCL8*, matrix metalloproteinases, and immediate early genes in 3D suggests a TME in which epithelial cells interact with adjacent cells; actively sensing and responding to signals from the localized microenvironment. In support of this, previous reports have identified inflammation and immune regulators as top DEGs in 3D versus 2D cultures of epithelial ovarian cancer cell lines (Kerslake et al., 2023). Notably, key upregulated genes including *C3*, *CXCL8*, *SLPI*, *CXCL1*, *CXCL2*, *IL1B*, *CCL20*, and *CFB*, in our 3D model were also upregulated in 3D ovarian cancer cell lines (Kerslake et al., 2023). Furthermore, top structural genes such as *PI3*, *FN1*, and *MMP1* similarly exhibited increased expression in both cervical and ovarian 3D models (Kerslake et al., 2023). The consistent enrichment of inflammatory mediators and ECM components highlights the improved ability of 3D cultures to recapitulate physiologically relevant tumor microenvironments across multiple cancer cell models.

IL-8 (*CXCL8*) is a key player in the interplay between hypoxia and dimensionality in the TME (Waugh and Wilson, 2008; DelNero et al., 2015). Additionally, elevated IL-8 levels have been linked to poorer survival in cervical cancer patients (Fujimoto et al., 2000). Clinical observations also support the notion that solid tumors exhibit heightened IL-8 signaling (Waugh and Wilson, 2008; DelNero et al., 2015). We observed higher expression of *CXCL8* in 3D cultures, suggesting culture dimensionality regulates tumor cell hypoxia response and angiogenesis. Multiple reports also suggest that IL-8 synergistically enhances *VEGF*-mediated angiogenesis (Hou et al., 2014; Perez-Gutierrez and Ferrara, 2023). In our study, both *VEGFA* and *VEGFB* were upregulated in 3D cultures. Collectively, this suggests that 3D culture conditions may promote angiogenic signaling by augmenting the synergistic interplay between VEGF and IL-8 signaling.

The abundant biological processes are in consensus with the IPA results, exhibiting enrichment of pathogen-induced cytokine storm, macrophage activation, and TME pathways. Meanwhile, downregulated GO biological processes were associated with tissue development and cell differentiation, such as myofibril and cartilage development, muscle structure morphogenesis, and epithelial differentiation. IPA analysis similarly showed upregulation of tissue remodeling and wound healing pathways. Further, the abundance of molecular functions in GO aligned with the IPA results showing increased matrix metalloproteinases and cytokines involved in tissue remodeling and inflammatory signaling. The IPA analysis similarly pointed to modulation of growth factors, ECM components, and wound healing. Further in consensus with the IPA results, GO molecular function analysis demonstrated increased peptidase/protease and inflammatory signaling activities and decreased binding interactions related to structural extracellular matrix and cytoskeletal components. Collectively, these in-depth analyses offer comprehensive insight into pathways and molecular mechanisms underlying the transcriptomic differences exhibited by 3D versus 2D SiHa cell cultures.

Dysregulation of pregnancy-specific glycoproteins has been consistently linked to colorectal and breast cancers (Moore et al., 2022). In cervical cancer, the *PSG2* locus harbors copy number gain associated with increased expression in cancer compared to normal from bulk tissue biopsies (Marrero-Rodriguez et al., 2018). Further, using micro-dissected tissues from normal, precancerous, and cancerous cervix, our group has previously shown decreasing stromal cell PSG expression with increasing cervical neoplastic stages (Gius et al., 2007). Investigating the regulation of PSGs in 3D cervical cancer cell lines along with single cell expression studies could further tease out the role of PSGs and their role in immune modulation in cervical cancer.

DEGs specific to culture condition and independent of HPV demonstrated upregulated genes associated with neutrophil degranulation (*ALDOC*, *ARHGAP45*, *PECAM1*, *S100A9*, and *SLPI*) and T-cell receptor signaling (*HLA-DQB1*, *HLA-DRA*, *HLA-DRB1*), suggesting enhanced immune signaling. The increased *S100A9* expression in our 3D spheroids aligns with previous findings that tumor-infiltrating monocytes/macrophages upregulate *S100A9* in cancer cells to promote invasion and migration (Lim et al., 2016). Furthermore, the upregulation of *CXCL8* (*IL-8*) and *CXCL2* observed in our 3D spheroid cultures may contribute to the recruitment and accumulation of tumor-associated neutrophils within TME. As tumor cells create a supportive niche to promote their survival, they can trigger an inflammatory response, consequently attracting more neutrophils into the TME (Wei et al., 2021). Elevated neutrophil infiltration into primary tumors has been associated with poorer prognosis and increased resistance to drug therapies in various cancers (Arvanitakis et al., 2021; Kim et al., 2022; Tian et al., 2022). Likewise, the increased expression of HLA class II genes, which play a role in T-cell receptor signaling, has been observed across various tumor types, including cervical cancer (Esteban et al., 1990; Hilders et al., 1993). The HLA class II proteins function as regulators of immune responses by presenting antigenic peptides to CD4^+^ T cells and by controlling B-cell differentiation into antibody-producing blasts (Seliger et al., 2017). While constitutive HLA class II antigen expression has been reported to be associated with a favorable prognosis in some tumor types, such as colorectal cancer and larynx squamous cell carcinoma (Esteban et al., 1990; Lazaris et al., 1995; Kunihiro et al., 1998), it has also been associated with higher metastatic dissemination, increased tumor stage, and reduced patient survival in other malignancies, such as melanoma and cervical carcinoma (Pollack and Livingston, 1985; Hilders et al., 1993; Seliger et al., 2017). Therefore, upregulation of HLA class II genes in our SiHa 3D model may have implications for tumor progression and immune evasion. Interestingly, upstream regulator analysis predicted activation of hypoxia-related pathways in 3D spheroids, including *HIF1A* and inhibition of *EGLN1* (involved in HIF-1α degradation). The activation of HIF and inhibition of *EGLN1* suggest a more hypoxic environment in the 3D spheroids, which may better mimic the known hypoxic conditions found in HPV-related cervical cancers. This highlights the potential advantages of using 3D spheroid models to study the tumor microenvironment.

HPV16 viral genes *E1*, *E2*, *E6*, and *E7* were expressed at significantly higher levels in cell lines grown in non-scaffold 3D versus 2D cultures, using identical media. A previous report showed increased HPV *E6* and *E7* gene expression in SiHa cells grown in fibroblast embedded ESM as compared to 2D monolayer cells (De Gregorio et al., 2020), which could partially contribute to heightened immune response signaling in 3D culture conditions. In this study, we also report a list of 26 host genes strongly correlated with viral gene expression across the 2D and 3D culture conditions. These genes are key players in cell cycle regulation and cancer, including *CDK3* and *COL7A1* (Zheng et al., 2008). The HPV DNA replication protein E1 has been previously reported to serve as a substrate for CDK complexes (Ma et al., 1999). Phosphorylation of E1 by CDKs is crucial not only for effectively activating HPV replication, but also for regulating its nuclear localization to facilitate viral DNA amplification (Deng et al., 2004). Notably, our results suggest that the 3D culture model may better mimic conditions that enhance HPV gene expression, possibly due to the interaction between CDKs and the viral replication-initiation protein E1.

Two other genes highly correlated with HPV gene expression, *S100A9* and *PLOD1*, are involved in inflammation and tissue remodeling and important for tumor growth, invasion, and metastasis (Lim et al., 2016; Wang et al., 2023). The significant role of large (*RPL10*, *RPL23*, *RPL13P12*, and *RPL21*) and small (*RPS27* and *RPS13*) ribosomal proteins in translation, a fundamental process for cell growth and division, is widely recognized. Meanwhile, *E2* is well-known for its role in initiating DNA replication and regulating transcription of HPV-encoded genes, notably the *E6* and *E7* oncogenes (Evande et al., 2023). Therefore, the strong correlation between ribosomal genes and HPV genes clearly emphasizes the complexity of HPV interactions with host cellular components. As part of the AP-1 complex, transcription factors *JUN* and *FOS* regulate the expression of both HPV viral genes and many host genes implicated in cancer development (Mittal et al., 1994; Chen et al., 1995). Indeed, reports have indicated the regulation of c-jun and c-fos by HPV oncoproteins (Mittal et al., 1994; Chen et al., 1995). Expression of these HPV-correlated genes in 3D models is particularly relevant because spheroids better mimic the *in vivo* cervical TME. This 3D model may allow for more accurate representation of gene expression patterns influenced by HPV infection, such as viral-induced proliferation genes (e.g., *CDK3*, *JUN*, *FOS*; Cho et al., 2009; Morgan et al., 2021) and HPV-mediated stress response genes (e.g., *IGFBP3*, *ZC3H12A*; Baege et al., 2004; Castagnino et al., 2022). Differential expression of these HPV-correlated genes between 2D and 3D models highlights the importance of using physiologically relevant systems for understanding virus-host interactions and developing novel HPV-targeted therapeutic strategies. Using a newly developed, patient-derived cervical cancer cell line, we further validated upregulated expression of HPV *E6* and *E7* oncogenes and the top five host genes showing positive correlation with HPV gene expression identified in 3D SiHa. Among these, the *ANKZF1* gene encodes a protein that interacts with p97, a key regulator of protein quality control, cell cycle progression, autophagy, and apoptosis (Lando et al., 2009; Huang et al., 2023). Knockdown of *ANKZF1* was recently shown to inhibit proliferation, migration, and invasion of colon cancer cells (Chen et al., 2023) and its association with poor survival has been reported in colorectal cancer (Zhou et al., 2019; Sajadi et al., 2022). *S100A9* has been shown to upregulate HPV oncogene expression by binding to the viral long control region and activating the transcription factor TEAD1 (Gius et al., 2007; Rader et al., 2008; Mori et al., 2023). However, Western blot analysis revealed that S100A9 protein was only highly expressed in the MCW-3 cell line and was not detected in SiHa 3D cultures. This discrepancy suggests the possibility of post-transcriptional regulation or cell line-specific variations in expression. We also validated 3D culture upregulation of *IGFBP3*, which has been reported to dramatically increase (500-fold) during HPV-mediated immortalization of cervical epithelial cells, with high levels secreted by late passage immortalized cells (Baege et al., 2004). In cervical cells, increased *IGFBP3* expression sensitizes cells to *IGF-1*, thereby enhancing IGF signaling, DNA synthesis, and proliferation.

Further, we performed protein-level validations for 3D-2D culture associated genes (*CASP14*, *S100A9*, *ALPP, CLDN1,* and *IL18*) using Western blot. These protein-level validations largely corroborate our transcriptomic findings, except *S100A9*, reinforcing the biological relevance of the identified gene expression changes in 3D versus 2D cultures. The consistency between transcript and protein levels for most examined genes in both cell lines underscores the robustness of our 3D culture system in modeling the complexities of the TME.

Overall, our transcriptomic analysis revealed upregulation of genes associated with immune response, angiogenesis, hypoxia, and ECM remodeling in 3D cervical cancer cell cultures. Accordingly, this aligns with a more physiologically relevant TME, coupled with higher HPV viral gene expression, suggesting these 3D models better represent HPV-related cervical cancers.

## Limitations – future work

While 3D cell cultures offer a more physiologically relevant representation of epithelial architecture and signaling pathways compared to conventional 2D cultures, they still fall short of replicating the intricate complexity of the TME observed *in vivo*. Our 3D model consists solely of epithelial cells, lacking the crucial interactions with other cell types that are typically present within the TME. Future research will be invaluable for improving the 3D model by incorporating non-epithelial cell types, such as immune cells and/or fibroblasts, into organoid cultures to better simulate cellular interactions in the tumor milieu. Our intent was to identify DEGs impacted by structure and not media or matrix. However, incorporating relevant ECM components into future 3D models could further resemble the native tumor environment. Moreover, comparing transcriptomic profiles of our 3D cultures to the original patient tumor, ideally with additional replicates, could unveil genomic similarities and differences that may have been overlooked during 2D adaptation. This comparison could further validate 3D models as more faithful representations of the *in vivo* tumor.

## Conclusion

The results of this study suggest that 3D cultures are valuable systems for studying HPV-mediated carcinogenesis and genes identified may represent potential targets for therapeutic intervention. Our 3D SiHa model exhibits a transcriptomic profile consistent with HPV oncogene activation coupled to inflammation, immune activation, and tissue remodeling. This represents a significant improvement over conventional 2D cultures, which lack the complexity to appropriately model TME biology. The 3D system provides a better platform to explore HPV immune evasion mechanisms, screen antiviral therapeutics, and understand host-virus interactions underlying cervical cancer progression.

## Data Availability

The datasets presented in this study can be found in online repositories. The names of the repository/repositories and accession number(s) can be found below: https://www.ncbi.nlm.nih.gov/geo/, GSE270674.

## Supplementary material

SUPPLEMENTARY FIGURE S1 Experimental design and morphology of 2D and 3D cell culture. **(A)** The experimental design: 2D samples were generated from 3 consecutive passages and from each passage 2 replicates were subjected to RNAseq. 3D samples were generated from non-consecutive passages. **(B)** Microscope images of SiHa cells grown in 2D culture. Images were acquired at ×4 magnification 3 days after initial plating. Scale bar = 50 μm. **(C)** Microscope image (20X) of a 5,000-cell SiHa spheroid 3D culture.

SUPPLEMENTARY FIGURE S2 A comprehensive overview of the significantly enriched core pathways using IPA, based on canonical pathways, upstream regulators, and functions/diseases. Pathways or networks with absolute Z-score ≥2 and *p-adj* < 0.05 were considered significantly enriched.

SUPPLEMENTARY FIGURE S3 Gene Ontology (GO) enrichment analysis of significantly DEGs in 3D vs. 2D culture conditions. List of top 10 biological processes **(A)** and molecular functions **(B)** which significantly exhibited higher and lower expression in 3D, with L2FC cutoff of ±1.5 and FDR of 0.05 based on GeneRatio (GeneRatio = k/n, where k is the overlap between genes-of-interest and the geneset, while n is the number of all unique genes-of-interest).
