# Supplementary figures and images for: Deciphering the divergent transcriptomic landscapes of cervical cancer cells grown in 3D and 2D cell culture systems
Source: Front Cell Dev Biol. 2024 Aug 13;12:1413882. doi: 10.3389/fcell.2024.1413882 (PMC11347336; doi:10.3389/fcell.2024.1413882)

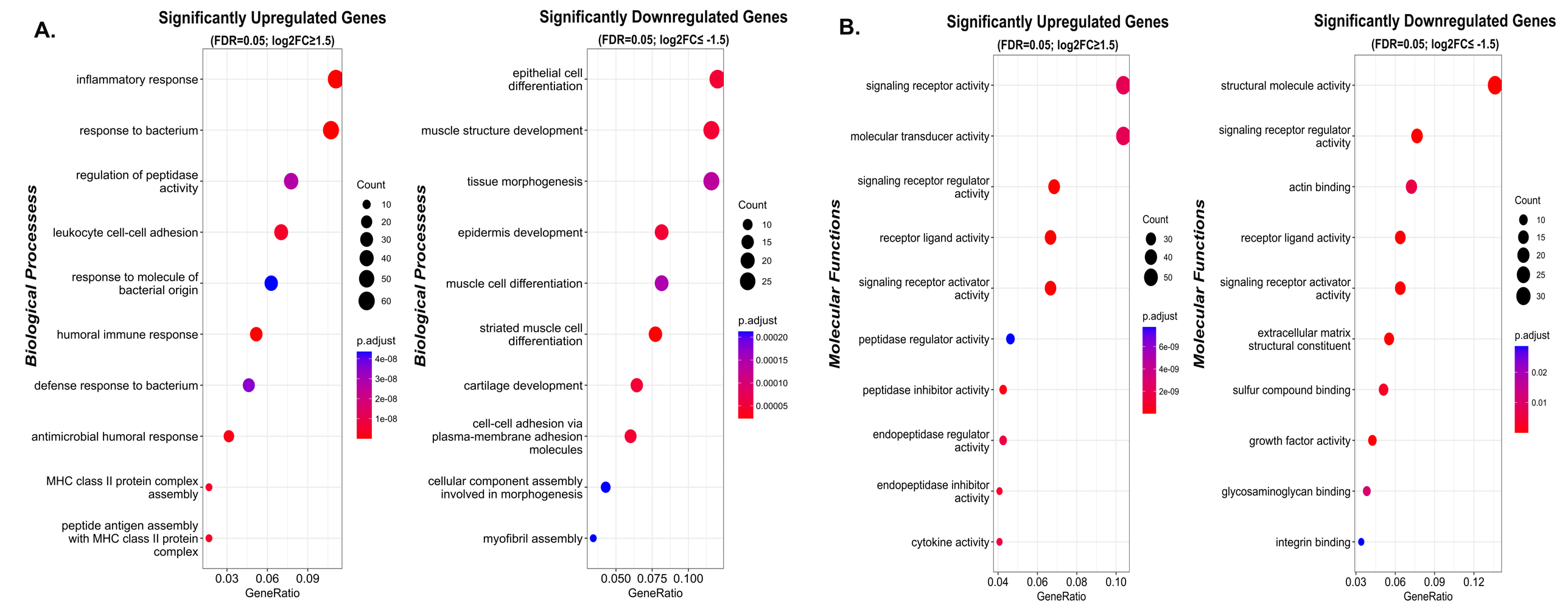

Supplement: Supplementary file 3 [file Image3.TIF]

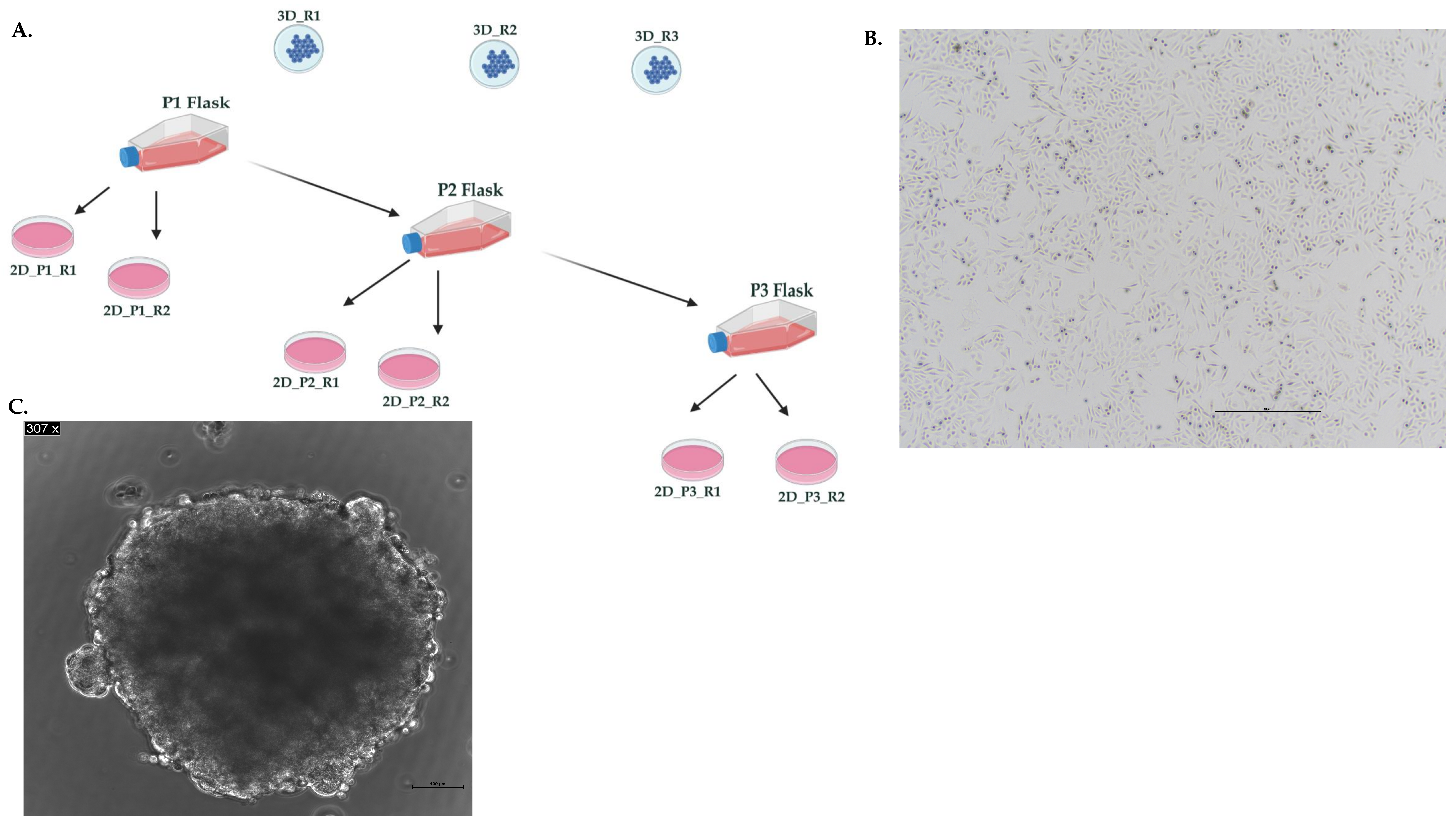

Supplement: Supplementary file 5 [file Image1.TIF]
